# The immune-metabolic crosstalk between CD3^+^C1q^+^TAM and CD8^+^T cells associated with relapse-free survival in HCC

**DOI:** 10.3389/fimmu.2023.1033497

**Published:** 2023-02-09

**Authors:** Yanying Yang, Lu Sun, Zhouyi Chen, Weiren Liu, Qiyue Xu, Fangming Liu, Mingyue Ma, Yuwen Chen, Yan Lu, Hao Fang, Geng Chen, Yinghong Shi, Duojiao Wu

**Affiliations:** ^1^ Jinshan Hospital Center for Tumor Diagnosis & Therapy, Jinshan Hospital, Fudan University, Shanghai, China; ^2^ Shanghai Key Laboratory of Bioactive Small Molecules, Department of Physiology and Pathophysiology, School of Basic Medical Sciences, Fudan University, Shanghai, China; ^3^ Shanghai Key Laboratory of Lung Inflammation and Injury, Institute of Clinical Science, Zhongshan Hospital, Fudan University, Shanghai, China; ^4^ Department of Liver Surgery and Transplantation, Liver Cancer Institute, Zhongshan Hospital, Fudan University, Shanghai, China; ^5^ Key Laboratory of Carcinogenesis and Cancer Invasion of Ministry of Education, Chinese Academy of Medical Sciences, Shanghai, China; ^6^ Research Unit of Bench and Clinic Research for Liver cancer Recurrence and Metastasis, Chinese Academy of Medical Sciences, Shanghai, China; ^7^ Center for Bioinformatics and Computational Biology, Shanghai Key Laboratory of Regulatory Biology, Institute of Biomedical Sciences, School of Life Sciences, East China Normal University, Shanghai, China; ^8^ Department of Endocrinology and Metabolism, Zhongshan Hospital, Key Laboratory of Metabolism and Molecular Medicine, the Ministry of Education, Fudan University, Shanghai, China; ^9^ Department of Anesthesiology, Zhongshan Hospital, Fudan University, Shanghai, China

**Keywords:** immunometabolism, C1q, tumor-associated macrophage, T cell, HCC

## Abstract

**Introduction:**

Although multiple targeted treatments have appeared, hepatocellular carcinoma (HCC) is still one of the most common causes of cancer-related deaths. The immunosuppressive tumor microenvironment (TME) is a critical factor in the oncogenesis and progression of HCC. The emerging scRNA-seq makes it possible to explore the TME at a high resolution. This study was designed to reveal the immune-metabolic crosstalk between immune cells in HCC and provide novel strategies to regulate immunosuppressive TME.

**Method:**

In this study, we performed scRNA-seq on paired tumor and peri-tumor tissues of HCC. The composition and differentiation trajectory of the immune populations in TME were portrayed. Cellphone DB was utilized to calculate interactions between the identified clusters. Besides, flow cytometry, RT-PCR and seahorse experiments were implemented to explore potential metabolic and epigenetic mechanisms of the inter-cellular interaction.

**Result:**

A total of 19 immune cell clusters were identified and 7 were found closely related to HCC prognosis. Besides, differentiation trajectories of T cells were also presented. Moreover, a new population, CD3+C1q+ tumor-associated macrophages (TAM) were identified and found significantly interacted with CD8+ CCL4+T cells. Compared to the peri-tumor tissue, their interaction was attenuated in tumor. Additionally, the dynamic presence of this newly found cluster was also verified in the peripheral blood of patients with sepsis. Furthermore, we found that CD3+C1q+TAM affected T cell immunity through C1q signaling-induced metabolic and epigenetic reprogramming, thereby potentially affecting tumor prognosis.

**Conclusion:**

Our study revealed the interaction between CD3+C1q+TAM and CD8+ CCL4+T cells and may provide implications for tackling the immunosuppressive TME in HCC.

## 1 Introduction

With a high mortality rate, liver cancer is the second leading cause of cancer-related deaths under the age of 80 worldwide (1, 2). Hepatocellular carcinoma (HCC) accounts for 70% to 85% of primary liver cancers (3). HCC is an inflammation-driven disease. Surgery, radiofrequency ablation (RFA), transcatheter arterial chemoembolization, and targeted therapies are the most common treatments (4). The tumor microenvironment (TME) of HCC is strongly immunosuppressive; thus, it is extremely important to illustrate the immune characteristics of TME and develop new immunotherapies for HCC.

Immune cell infiltration is a well-known significant regulator of HCC progression (5). The density of tumor-infiltrating CD8^+^T cells was proved to be an effective prognostic indicator in HCC or many solid tumors (6, 7). It can be affected by multiple regulatory procedures in HCC TME, including the secretion of transforming growth factor β (TGF-β) and interleukin 10 (IL-10), the recruitment of regulatory T cells (Tregs) and myeloid-derived suppressor cells (MDSCs), high levels of programmed cell death 1 (PD-1), programmed death ligand-1 (PD-L1) and so on (4, 5, 8, 9). All the factors cause a rather low response rate of HCC to immunotherapy (10). Therefore it is critical to illustrate the complex network of cell-to-cell interactions within the TME. However, the traditional immunological technology has certain limitations. For example, the chasm between *in vitro* and *in vivo* systems, low throuhput, and limited information on immunohistochemistry, et al.

ScRNA-seq is an emerging and powerful tool for investigating the cellular components even rare populations and their interactions in TME (11, 12). It also helps to illustrate connections between TME and clinical outcomes in cancers (13–15). Recently several studies have portrayed the landscape of HCC at single-cell level (16–20). For example, heterogeneity of exhausted T cells (Tex) has been reported (16, 17). The study aims to deepen our understanding of cell-cell interactions and molecular pathways in TME based on scRNA-seq data of paired HCC tissues, and discover new cell subsets which cannot be achieved by traditional methods (21, 22).

We revealed TILs differentiation trajectories and identified certain populations associated with HCC prognosis. The scRNA-seq found that macrophage populations were much more complex than the M1/M2 dichotomy. Although macrophages and T cells are generally considered to belong to different cell lineages, recently a novel macrophage sub-population expressing CD3 molecule was reported in infectious, inflammatory diseases (23). However, the specific role of CD3^+^ tumor-associated macrophages (TAM) is poorly understood. In the study, we found that a new population of CD3^+^C1q^+^TAM regulated the anti-tumor immunity of the tumor-infiltrated CD8^+^CCL4^+^T cells through the C1q signaling pathway and subsequent metabolic and epigenetic remodeling, thereby potentially affecting tumor prognosis. Our study supported that the versatile molecular C1q expressed in TAM has functions beyond the complement cascade. The data reveals the interaction between C1q and the metabolism of CD8^+^T cells and provides implications for regulating immunosuppressive TME.

## 2 Methods

### 2.1 Human specimens

Paired carcinomatous and para-carcinomatous tissues were from patients with HCC. No chemotherapy or radiation therapy was performed on patients before tumor resection. Peri-tumor tissue was 3cm away from the edge of the conjugated tumor tissue. Samples were then obtained for the subsequent CD3^+^cell sorting, single-cell RNA sequencing analysis, or *in vitro* testing implement.

Peripheral blood was collected from sepsis patients on the 1^st^, 3^rd^, 7^th^ and14^th^ day for flow cytometry. We obtained approval from the ethics committee of Zhongshan Hospital, Fudan University, and written informed consent from all HCC patients.

### 2.2 Sample preparation

We immersed specimens of fresh tumor and adjacent normal tissue in RPMI‐1640 (Gibco) containing 10% FBS, shredded, ground the blocks and then passed the suspension through 40 μm cell strainers. Next, the single cell suspension was centrifuged (1500 rpm, for 10 min) and the supernatant was removed. The bottom cell pellets were resuspended in erythrocyte lysis buffer (Solarbio), kept on ice for 5 min and then washed twice with 1 × PBS.

### 2.3 Cell isolation and scRNA-Seq

Single-cell suspension was stained with fluorescent-labeled anti- CD3 (0.5%, Biolegend, Cat No.300308, Clone HIT3a) anti-CD45RO (0.5%, Biolegend, Cat No.304210, Clone UCHL1) for 30 min at 4°C. Subsequently, cells were rinsed and resuspended for FACS sorting. CD3^+^CD45RO^+^ T cells were isolated through FACS sorting (BD FACS Aria II).

For scRNA-Seq, the sorted cells were counted with a hemocytometer and diluted to 700–1200 cells/μl with targeted cell viability (>70%). Single cells were separated on a Chromium controller (10×Genomics) as the manufacturer’s instructions. 20 cDNA libraries were prepared using Single-Cell 3′ Reagent Kits V2 (10xGenomics, Pleasanton, California) after single cell purification on a Chromium controller (10×Genomics) following the manufacturer’s instructions. Library sequencing was conducted *via* Illumina sequencer following stringent quality control by fragment analysis (AATI) and the output data was processed through the Cell Ranger pipeline (version 2.1.1, 10×Genomics) default. Cells expressing less than 200 genes or with an improperly high fraction (> 5%) of mitochondrial genes were removed. The raw data was normalized on a log scale, facilitating the following clustering and principal component analysis. CytoTRACE (https://cytotrace.stanford.edu) helped to predict cell differentiation fate based on single-cell RNA-sequencing data Cell development trajectory was performed through ScVelo, an extensible RNA velocity analysis toolkit.

### 2.4 CD8^+^ T cells culture

Spleens of wild-type mice were extracted, ground, and filtered to single-cell suspension. CD8^+^ T cells were purified through negatively magnetic sorting (Biolegend, Cat. 480008), activated with anti-CD3 (eBioscience, Cat.16-0031-85) and anti-CD28 (eBioscience, Cat.16-0281-85) for 3 days, and then cultured with 100 U/ml IL-2 (Peprotech, Cat.200-02-50) or 10 μg/ml IL-15 (R&D, Cat.247-ILB-005) for another 3 days while adding 10 ug/mL or 25ug/mL C1q (Sigma, Cat.C1740-1MG).

### 2.5 FACS analysis

For flowing staining, viable cells identified through Fixable Viability Stain 510(BD). And cells were stained with CD3 (BioLegend, clone: HIT3a), CD8 (BioLegend, clone: SK1), CD45 (BioLegend, clone: HI30), CD68 (BioLegend, clone: Y1/82A), CD11B (BioLegend, clone: ICRF44), CD80 (BioLegend, clone: 2D10), CD206 (BioLegend, clone: 15-2), CD163 (BioLegend, clone: RM3/1), C1QA (abcam, clone: EPR2980Y), C1QC (abcam, clone: EPR2984Y), PD-1 (BioLegend, clone: RMP1-30), PD-L1 (BioLegend, clone: 10F.9G2), BCL2 (BioLegend, clone: 100), Ki67 ((BioLegend, clone: 11F6), GZMA (Biolegend, clone: CB9),GZMB (Biolegend, clone: QA16A02), IFNγ (Biolegend, clone: XMG1.2), TNFα (Biolegend, clone: MP6-XT22), LDHA (biocompare), Acetyl-Histone H3 (Lys27) Antibody (Cell Signaling), Acetyl-Histone H3 (Lys9) (C5B11) Antibody (Cell Signaling). Intracellular markers were stained before cell re-stimulation by PMA/ionomycin (Biolegend, Cat. 423303). Flow cytometry was performed on BD FACS Aria III flow cytometer and the data was analyzed by FlowJo6 software.

### 2.6 Metabolic gene-expression analysis by RT-PCR

Relative expression levels of selected genes were quantified by qRT-PCR. cDNA was synthesized *via* PrimeScript RT Master Mix (TAKARA, Cat. RR036A). TB Green Premix Ex Taq II (TAKARA, Cat. RR820A) was used for qRT-PCR analysis on the Roche LightCycler 480 System with primer sets in **Supplementary Table 1**. Expression data of all candidate genes were normalized to the housekeeping gene *36b4.*

### 2.7 Metabolic assay

XF-96 extracellular flux analyzer (Seahorse Bioscience) was used for the mitochondrial fitness test, Oxygen consumption rate (OCR) was measured at basal (1.5 × 10^5 cells/well), and after treatment with oligomycin (1 μM), protonophore carbonyl cyanide p-trifluoromethoxyphenylhydrazone (FCCP,1.5 μM), etomoxide (200 μM) in some cases, and rotenone (100 nM) plus antimycin A (1 μM) sequentially. The lactate assay kit (Life Technologies) was used to measure the lactate content in cells and cell cultures.

### 2.8 Statistical analysis

Post-acquisition analysis was carried out on FlowJo Software (v10.5.30) and representative plots were exhibited. Statistical analysis and corresponding quantitative plots were conducted using GraphPad Prism 8 software. Significance levels were assessed by Student t-test or ANOVA test, where appropriate.

### 2.9 Data availability

Relevant data in this study are available within the article and supplementary files. Additional information is available upon reasonable request from the corresponding author

## 3 Results

### 3.1 Cell populations identification in HCC samples

**Figure 1:**
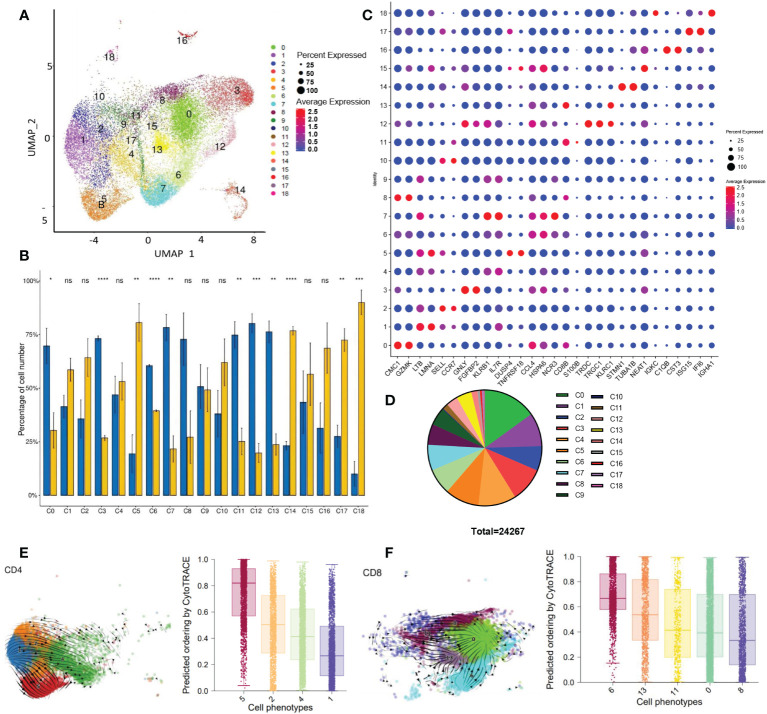
Single CD3^+^ cell transcriptome analysis **(A)** UMAP visualization of T cells clusters based on 24267 single-cell transcriptomes of 6 samples (paired tumor and peri-tumor tissue of HCC), showing the formation of 19 cell clusters. **(B)** Histogram visualization shows the tissue distribution of 19 clusters. The percentage of cell number of each cluster was compared between tumor and peri-tumor. The means of the 2 groups were tested for significant differences based on the *t-test*. ns, no significance; *P<0.05 **P<0.01; ***P<0.001; ****P<0.0001. **(C)** Dot plot showed the expression of representative genes for each cluster. **(D)** Pie charts showed the proportion of different clusters. **(E)** Developmental trajectories of CD4^+^T clusters in UAMP space by scvelo (right) and CytoTRACE (left). Values per phenotype using boxplots in CD4^+^ clusters. Scores from 0 to 1 represent progressively lower differentiation potential. **(F)** Developmental trajectories of CD8^+^T clusters in UAMP space by scvelo (right) and CytoTRACE(left). Summarize the median and distribution of CytoTRACE values per phenotype using boxplots in CD8^+^T clusters. Scores from 0 to 1 represent progressively lower differentiation potential.

To identify the cellular diversity in HCC patients, we implemented ScRNA-Seq using the 10x Genomics single-cell 3′ V2 chemistry. We generated a single-cell suspension of 6 samples from the paired tumor (T) and peri-tumor tissue (P) and enriched CD3^+^T cells by magnetic bead sorting. Barcoded sequencing reads went to the corresponding cells and transcriptome, then individual mRNA molecules were counted through unique molecular identifiers (UMIs). After quality control, we acquired single-cell transcriptomes of 24267 cells. Multiple single-cell analysis was carried out *via* R package *Seurat* (version 3.0; https://satijalab.org/seurat/) and high-quality transcriptome and visualization cells were presented over uniform manifold approximation and projection (UMAP) (**Figure 1A**). A total of 19 immune cell clusters were identified, including CD8^+^T cells (5 clusters) and CD4^+^T cells (4 clusters), NKT cells (2 clusters), gamma delta T cells, CD3^+^monocytes and some clusters undergoing proliferating or differentiating process, et al. (**Figure 1A**; **Table 1**). In addition to the typical ones, including naïve T cells (TN), central memory T cells (TCM), effector memory T cells (TEM), recently activated effector memory or effector T cells (TEMRA/TEFF, designated TEMRA hereafter), gamma delta T cells, tumor-Treg cells, and exhausted T (Tex) cells, we also identified two virus-responsive cell clusters, with the CD3-IFI6 and CD3-IGHA1 clusters expressing some markers of type I interferon-stimulated genes.

**Table 1:** The presentative genes of each cluster.

Cluster NO.	Name	Definition	Representative genes
0	CD8-CMC1	Central memory	CMC1, GZMK, CCL4, CCL3, CCL4L2
		CD8 T	
1	CD4-GPR183	Central memory CD4 T	LTB, LMNA, GPR183, VIM, IL7R
2	CD4-LEF1	Effector memory CD4 T	SELL, CCR7, LEF1, LDHB, GPR183
3	CD3-GNLY	NK	GNLY, FGFBP2, GZMB, GZMH, NKG7
4	CD4-CD69	TEMRA	KLRB1, IL7R, FOS, NFKBIA, CD69, CD40LG
5	CD4-FOXP3	Treg	FOXP3, TIGIT, IL2RA
6	CD8-CCL4	Effector CD8 T	CD8A, CCL4, HSPA6, FOSB, DUSP1, ID2
7	CD3-KLRB1	NKT	KLRB1, NCR3, CEBPD, CD69, DUSP1
8	CD8-GZMK	Central memory CD8 T	GZMK, CMC1, CCL5, CD8B, CD8A, NKG7
9	CD3-NCR3	NKT	NCR3, LTB, IL7R, KLRB1
10	CD3-SATB1	Differentiating T	MAL, LEF1, SATB1, CCR7, SELL
11	CD8-S100B	Effector memory	LEF1, S100B, CD8A, CD8B
		CD8 T	
12	CD3-TRGC1	Gamma delta T	TRDC, TRGC1, GNLY, KLRD1, KLRC1
13	CD8-KLRC1	Effector CD8T	KLRC1, CD8B, KLRC2, CCL5, XCL1, KLRD1
14	CD3-STMN1	Proliferating cells	STMN1, PCNA, MKI67
15	CD3-CTLA4	Exhausted T cells	NEAT1, FOSB, CTLA4, TIGIT, HAVCR2
16	CD3-CD68	TAM	C1QB, C1QA, C1QC, APOEFTL, SELENOP, FCER1G, HLA-DRA, CD68
17	CD3-IFI6	Virus responsive T	IFI6, ISG15, MX1, IFI44L, IFIT3
18	CD3-IGHA1	Undetermined	IGKC, LTB, IGHA1, IFI6

We found that 11 clusters were differentially distributed in P and T. Statistical analysis was performed on the number of cells in each cluster (**Figure 1B**). Except for the C8, other CD8^+^ T cell clusters (C0, C6, C11, C13) had a remarkable reduction in tumor tissue than adjacent tissue. Among the CD4^+^ T cell clusters, C5(Treg) was significantly accumulated in the tumors, centering in establishing and maintaining an immunosuppressive environment. We then listed representative genes for each cluster in **Figure 1C**. Each cluster exhibited a distinct distribution and characteristics. From C0 to C9, the top 10 clusters of most CD3^+^cells infiltrated in HCC samples are mainly composed of CD4 and CD8 T cells (**Figure 1D**). Other cell types, C3(NK) C7(NKT), and C12 (Gamma delta T) are notably reduced in tumors; on the contrary, the proportion of C14: CD3-STMN1 (proliferating cells), C17: CD3-IFI6 (type I interferon signaling pathway activated T cells) increased significantly in the tumor.

### 3.2 Time differentiation trajectory map of different cluster cells

Next, we explored a hypothetical differentiation trajectory with multiple intermediate states and gene expression gradients by using scVelo and CytoTRACE. The scVelo and CytoTRACE analysis respectively reflect the direction and potential of cell differentiation.

Firstly, we obtained the developmental trajectory of CD4^+^ and CD8^+^ T cells on UAMP space *via* scVelo. As listed in **Table 1**, we identified 4 clusters of CD4^+^T cells. It was found that C1 showed progress trends toward 3 other clusters (**Figure 1E**). Using CytoTRACE to predict the differentiation potential of 4 clusters, in order from high to low is C1> C4> C2> C5(from purple to red, **Figure 1E**; **Supplementary Figure 1A**), which is consistent with the results of scVelo. The results showed that C1 (GPR183^hi^IL7R^hi^CD4^+^T cells) was speculated to be a group of cells with “stem cell-like” multiple differentiation potential. The memory CD4 T C2 with up-regulated expression of SELL, CCR7, and LEF1, may differentiate into effector CD4 (C4) or regulatory T cells (C5). C4 has TEMRAs. C4 and C5 developed into two individual branches without further differentiation potentials. The top 10 genes related to stemness (red) or differentiation (blue) were listed in **Supplementary Figure 1B** by calculating the correlation with CytoTRACE. It is not surprising that multiple genes coding ribosome proteins were related to CD4^+^T cell differentiation (**Supplementary Figure 1B**, blue part). Ribosome biogenesis is critical for T-cell activation because of its rate-limiting role in cell growth and proliferation (24). More interestingly, several immune checkpoint genes (TIGIT, CTLA4) were linked to stemness character (**Supplementary Figure 1B**, red part), reflecting a paradox that dysfunctional TILs *in situ* may be capable of stem cell-like behavior (25).

By analyzing the differentiation trajectory of CD8^+^T cells using the same strategy, scVelo depicted that the differentiation direction started from C11 to other clusters (**Figure 1F**). CytoTRACE predicted that the differentiation potential of the clusters varied from high to low by C8> C0> C11> C13> C6 (**Figure 1F**, purple to red). Track analysis showed the same trends (**Supplementary Figure 1C**). Different from the results of CD4^+^T cells, multiple genes coding ribosome proteins were linked to cell differentiation and stemness of CD8^+^T cells (**Supplementary Figure 1D**, blue part, and red part respectively), pinpointing the importance of metabolism in CD8^+^T cells. Besides, other genes including long non-coding RNA were found associated with CD8^+^T cell differentiation (**Supplementary Figure 1D**, blue part), suggesting diverse factors are required for the process.

### 3.3 Survival analysis of each cluster for liver cancer patients

**Figure 2:**
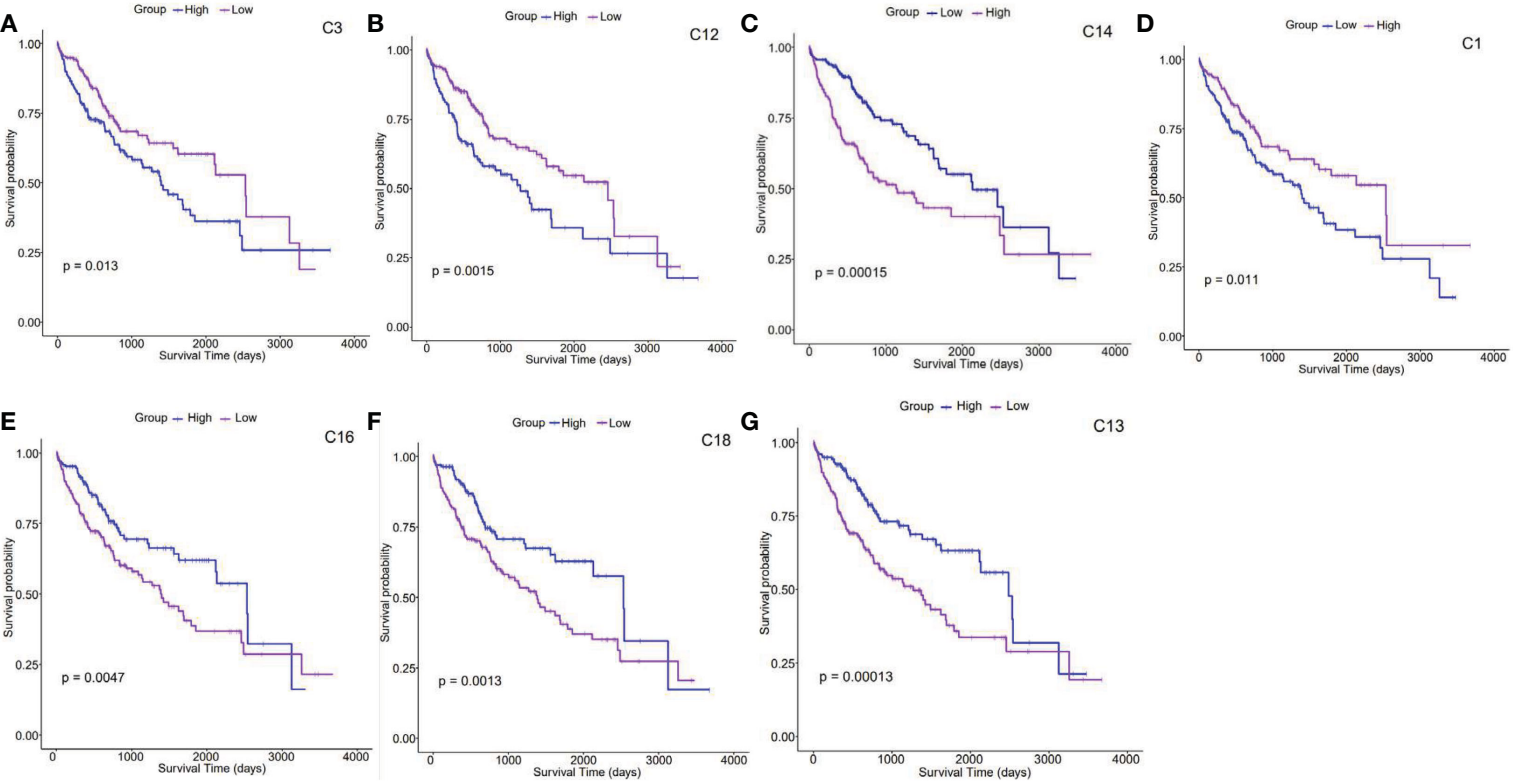
Survival analysis of clusters **(A–G)**. Kaplan–Meier survival analysis curves compare the high and low expression of characteristic genes in each cluster based on the TCGA HCC cohort. .

Based on the TCGA data of 368 liver cancer patients, the survival analysis results obtained 7229 genes related to liver cancer prognosis. In each cluster, we counted the number of survival-related genes. It was found that C14 had the most survival-related genes, followed by C16, and C18, while there were no survival-related genes in C9, C10, and C11. To further understand the role of different cell subsets in prognosis, we performed a K-means cluster analysis on TCGA data based on the survival-related characteristic genes of liver cancer for each cluster. We divided liver cancer patients into two groups and calculated the survival curve to judge the role of this cluster in predicting the prognosis of patients (**Figure 2**).

According to survival analysis results, survival curves of 7 clusters (C1, C3, C12, C13, C14, C16, and C18) could be significantly separated. Patients highly expressing C3, C12, and C14-featured genes presented a lower survival probability than those with moderate levels, indicating that the prognosis of patients with liver cancer enriched with this type of cells *in vivo* is relatively poor (the upper panel**, Figures 2A–C**). On the contrary, those with high expression ofC1, C13, C16, and C18-featured genes showed improved survival probability, suggesting that these clusters may point to better prognosis in liver cancer patients (the lower panel, **Figures 2D–G**). Interestingly, cell cycle genes STMN1 and TUBA1B were found increasingly expressed on C14. As switch molecules of the cell cycle from G1 to S phase, they were known for their crucial rules in eukaryotic cell proliferation (26). As shown in **Figure 1B**, the proportion of C14 increased by 3 times in HCC tumors. Although the destiny of these proliferating cells remains unknown, survival analysis suggests that it is detrimental to the prognosis of cancer patients (**Figure 2C**).

### 3.4 The interaction between clusters

It is essential to comprehend the interactions between clusters. Inter-cellular interactions by various molecules, such as receptors, ligands, structural proteins, secreted proteins, hormones, cytokines, neurotransmitters, etc, mediate cell communication and cell activities. Cellphone DB provides comprehensive recourses of curated receptors, ligands, and interactions(https://pypi.org/project/CellPhoneDB/).

**Figure 3:**
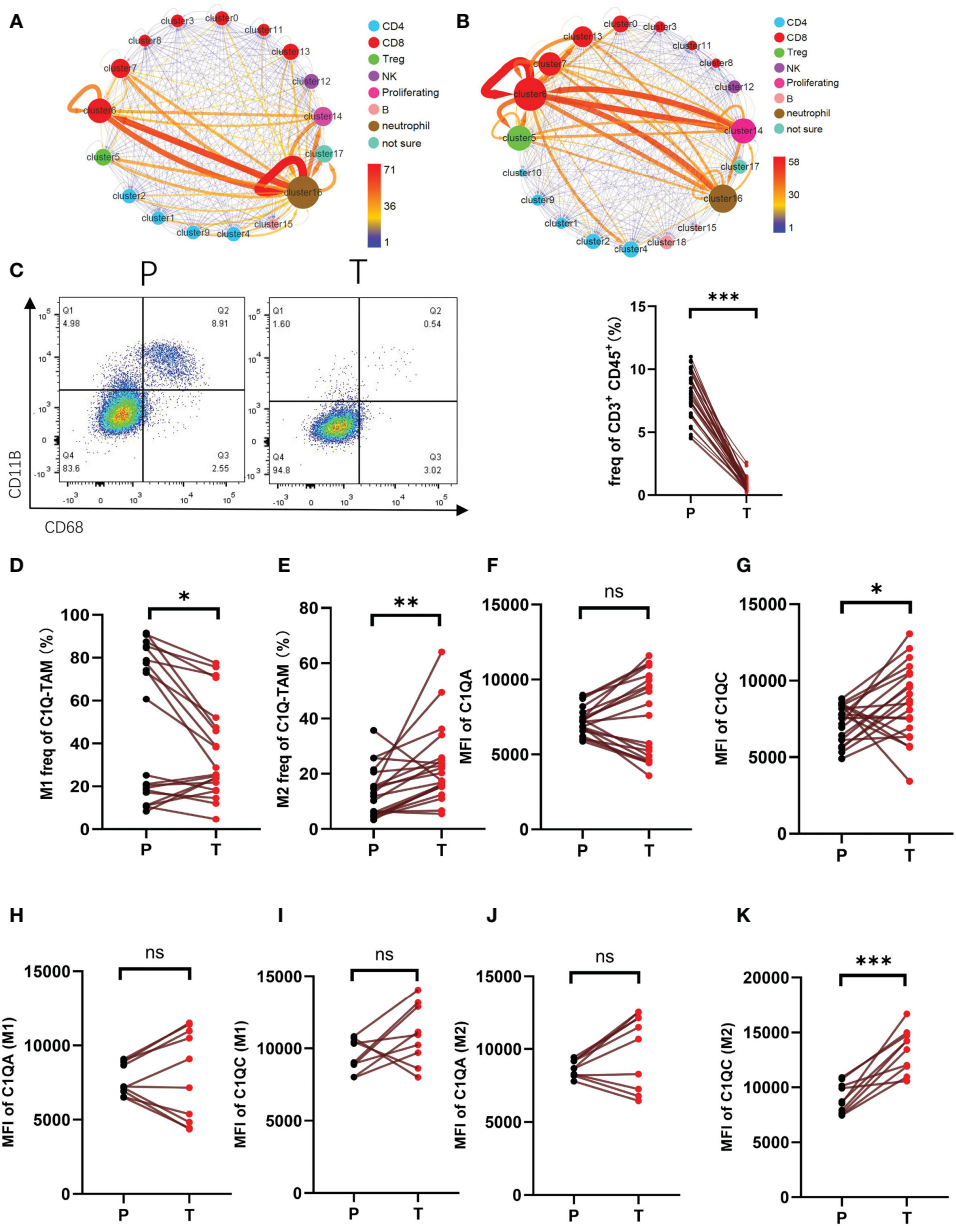
The interaction of CD3^+^C1q^+^TAM and CD8^+^CCL4^+^T cells a-b. Interaction between clusters of HCC para-cancer **(A)** and tumor tissues **(B)**. The arrow represents the interaction from this cluster to that cluster. The thickness of the line indicates the value of the interaction pair. The size of the point refers to the interaction of the cluster as an end. The color of the point refers to the interaction of the cluster as a start. **(C)** Flow cytometric gating of C1q-T (CD11B^+^ and CD68^+^) staining on CD45^+^CD3^+^ cells of HCC. Right panel shows the proportion of CD11B^+^ CD68^+^ in total CD45^+^CD3^+^ cells. **(D, E)**. The proportion of M1 (CD86^+^CD80^+^) **(D)**, M2 (CD163^+^CD206^+^) **(E)** in total C1Q-TAM (CD11B^+^ and CD68^+^) were compared at P and T. **(F–K)**. The expression level of C1QA and C1QC of C1Q-TAM cells **(F, G)**, M1 **(H, I)** and M2 **(J, K)** were detected by cell flow cytometry. ns, no significance; *P<0.05; **P<0.01; ***P<0.001.

In the study, we utilized scRNA-Seq data and Cellphone DB (version 3.1.0) to calculate the interaction scores between clusters, visualized the receptor-ligands interaction quantity, and analyzed the regulatory relationship. The output results are counted and presented in **Figures 3A, B**. **Figures 3A**, **B** respectively showed the interaction of clusters within peri-tumor and tumor tissue. We found that in HCC, the network with the most notable changes involved clusters C5, C16, and C4. In para-cancerous tissue, C6 and C16 had stronger interactions (**Figure 3A**). However, C16 not only lost self-regulation but also had a weaker connection with C6 in the tumor. In contrast, tumor-infiltrated C6 and C14 gained enhanced interactions (**Figure 3B**). Overall, the changed connections between clusters of TME suggested the potential immune suppressive mechanisms which need further investigation.

A meaningful receptor-ligand interaction leads to a physiological response. We identified 141 pairs of ligand-receptor interactions enriched in tumor samples. And the top five were KLRB1_CLEC2D, CD74_MIF, MIF_TNFRSF14, CD8 receptor_LCK, and CD2_CD58. There were 156 pairs in the samples of adjacent tissues, and the top five with the highest frequency were CD74_MIF, CD55_ADGRE5, MIF_TNFRSF14, SELL_SELPLG, CCL4L2_VSIR. The role of these ligand-receptor interactions in shaping TME still needs further investigation.

Next, we defined C16 as CD3^+^C1q^+^TAM. The presentative marker genes for C16 included the genes encoding the C1q protein family (C1QB, C1QA, C1QC, and CD68) (**Figure 1C**). The C6 was CD8^+^CCL4^+^T cells identified as effector T cells. Therefore, we speculate that C16 possibly regulates C6 function through the complement signaling pathway. The cluster C16 (CD3^+^C1q^+^TAM) found in our study has not been reported before. To further evaluate the existence of C16, we stain CD3^+^ CD45^+^ CD68^+^ CD11B^+^ cells for single-cell suspension of paired tumor and peri-tumor tissue (**Figure 3C**). Using flow cytometry, we detected the presence of CD3^+^C1q^+^TAM in HCC and found the proportion of this population was significantly lower in tumors (**Figure 3C**). The observation is consistent with the shift revealed by scRNA-Seq analysis (**Figures 3A, B**). Furthermore, we labeled M1-LIKE(CD86^+^CD80^+^) and M2-LIKE(CD206^+^CD163^+^) to explore its entity (**Supplementary Figure 2**). The staining suggested that the population CD3^+^C1q^+^TAM was a mixture of M1 or M2-LIKE cells. There are more M1-LIKE cells in tumor-adjacent tissues (**Figure 3D**). In contrast, a remarkably increased number of M2-LIKE cells was observed in tumor tissues (**Figure 3E**). Since the C1q signaling pathway was significantly expressed in C16 (**Figure 1C**). C1QC expression was elevated in tumors, especially in M2 cells, and C1QA did not change significantly (**Figure 3F–K**).

**Figure 4:**
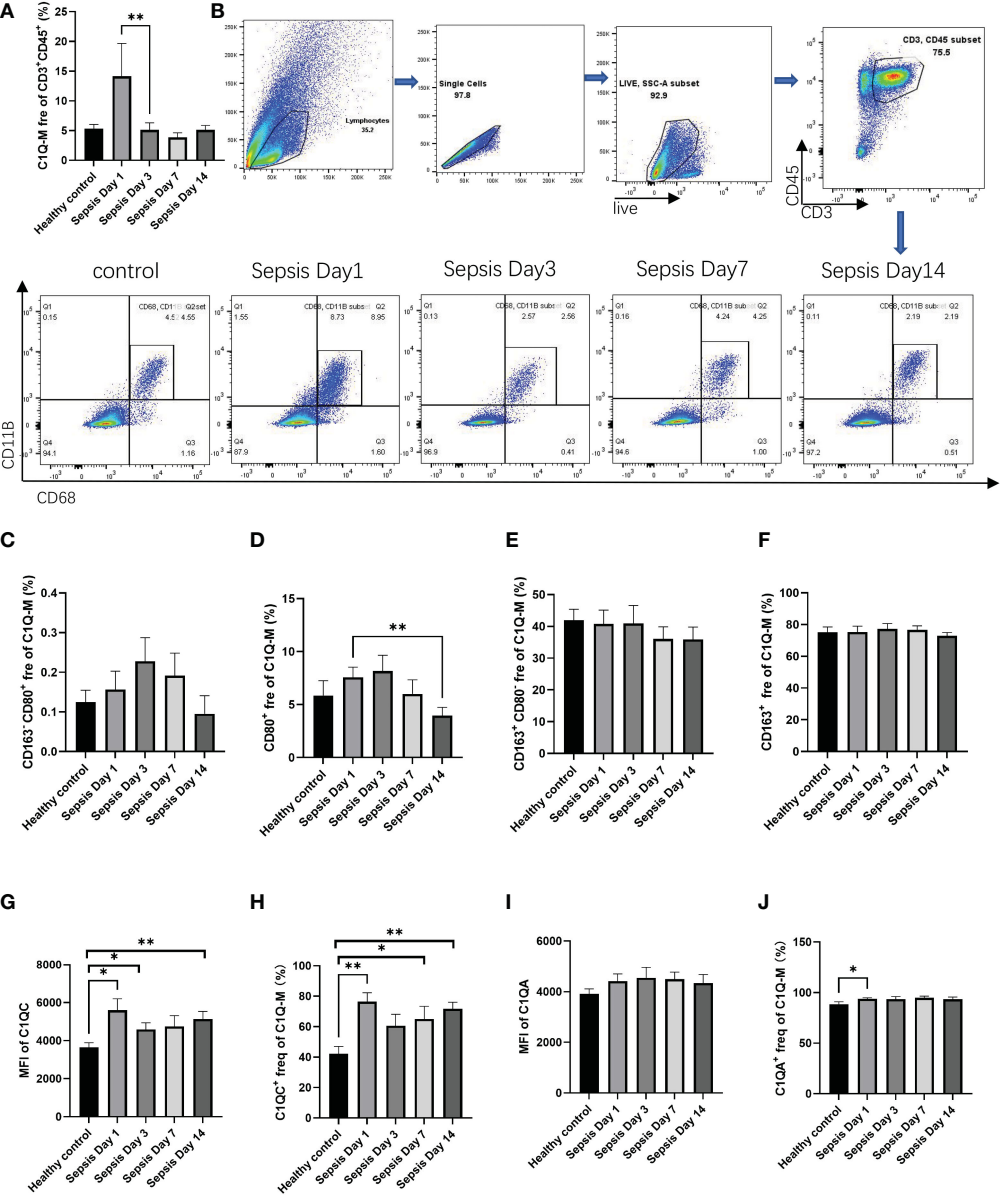
The existence of CD3^+^C1q^+^macrophage in peripheral blood of sepsis patients **(A)** The histogram shows the proportion of CD11B^+^CD68^+^ in the total CD45^+^CD3^+^ cells in the peripheral blood of healthy controls and sepsis patients. **(B)** Flow cytometry gating of C1Q-M (CD11B^+^ and CD68^+^) staining on CD45^+^CD3^+^ cells in healthy controls and sepsis. c-f. The histogram shows the proportion of CD163^-^CD80^+^ **(C)**, CD80^+^ **(D)**, CD163^+^CD80^-^ **(E)**, CD163^+^ **(F)** cells in total C1Q-M (CD11B^+^ and CD68^+^) by flow staining. **(G–J)** The expression levels **(G, I)** and ratios **(H, J)** of C1QA and C1QC in C1Q-TAM cells **(F–G)**, M1 **(H–I)** and M2 **(J–K)** were detected by flow cytometry. ns, no significance; *P<0.05; **P<0.01.

To further verify the existence of this population, we collected peripheral blood from patients with sepsis to measure the proportion of CD3^+^C1q^+^macrophage in infection by flow cytometry. We found that the CD3^+^C1q^+^macrophage ratio increased in the early stage of sepsis (day 1) and decreased after day 3 (**Figures 4A, B**). Furthermore, we labeled M1-LIKE (CD80^+^) and M2-LIKE (CD163^+^) and the data indicated that M2-LIKE cells were significantly higher than M1-LIKE cells in the peripheral blood of sepsis patients (**Figures 4C–F**). At the same time, we also detected the C1q signaling pathway and found that the expression of C1QC was elevated in the peripheral blood of patients with sepsis, while C1QA had no significant change (**Figures 4G–J**). These results are consistent with the HCC results, so we believe that CD3^+^C1q^+^TAM plays a pro-inflammatory and anti-tumor role in the initial stage of infection and tumors.

### 3.5 Effects of C1q on T cell metabolism

**Figure 5:**
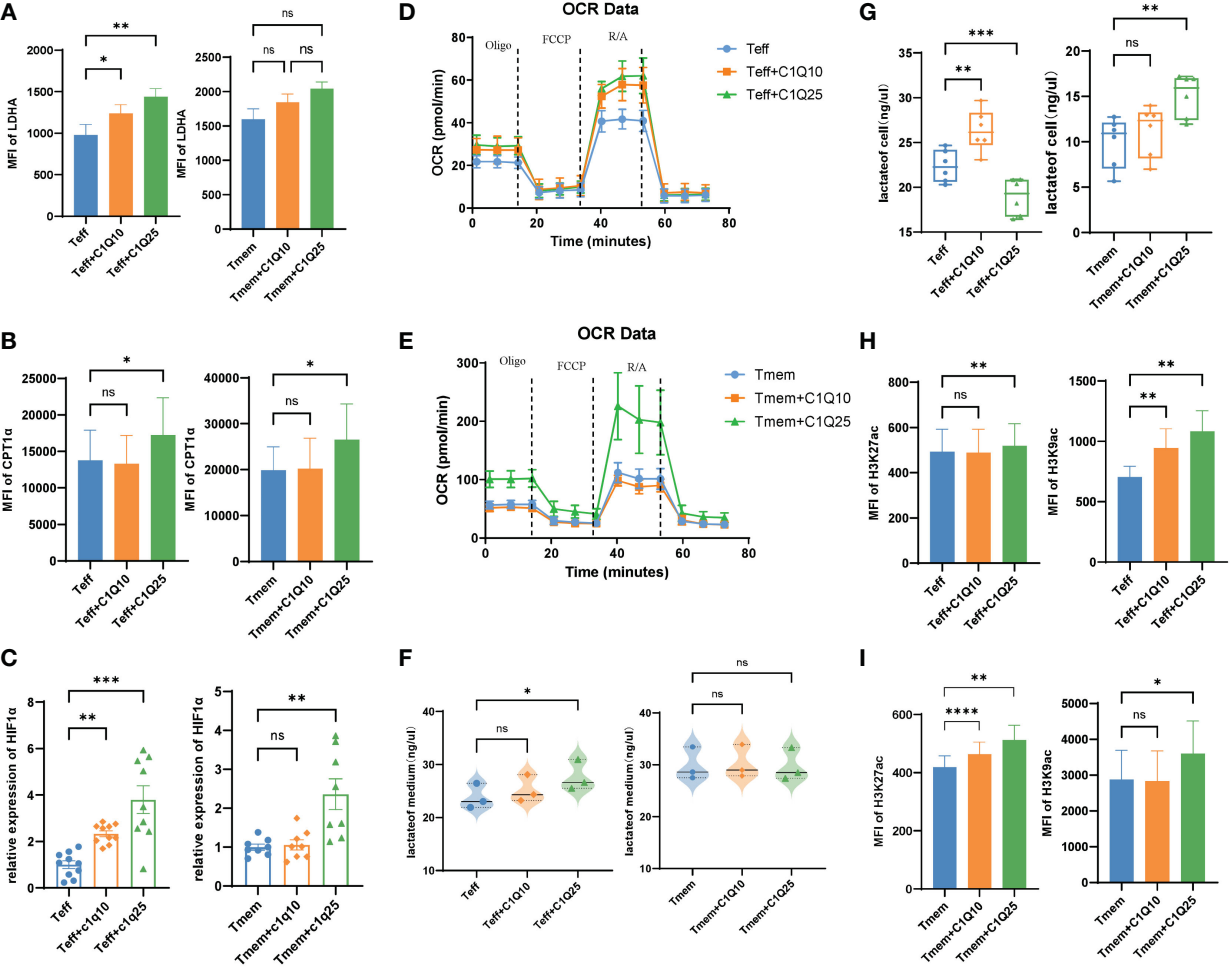
Effects of C1Q on T cell differentiation and function **(A)** The expression of LDHA in Teff and Tmem cells with and without C1q. **(B)** The expression of CPT1α in Teff and Tmem cells with and without C1q. **(C)** The mRNA expression of HIF1a in Teff and Tmem cells with and without C1q. **(D, E)**. Oxygen consumption rate (OCR) spectra of Teff and Tmem cells with and without C1q using Seahorse XF96 analyzer. **(F)** The lactate levels in Teff and Tmem cells with and without C1q. **(G)** The lactate levels in Teff and Tmem cells culture medium. **(H)** The expression of H3K27ac in Teff and Tmem cells with and without C1q. **(I)** The expression of H3K9ac in Teff and Tmem cells with and without C1q. ns, no significance; *P<0.05; **P<0.01; ***P<0.001; ****P<0.0001.

Since energy metabolism is essential for T cell differentiation and function, we next explored how C1q modulates T cell metabolism. We tested the expression panel of metabolic genes and found most were upregulated in C1q-treated Teff cells, including HK1, TPI1, GPD2, GPM2, ENO3, PDK1, and LDHA (**Supplementary Figure 3**). In Tmem cells, only the expression of ALDOA and LDHA were increased after C1q treatment (**Supplementary Figure 3**). For both Teff and Tmem, the MFI of Carnitine palmitoyl transferase 1A(CPT1α) and Lactate dehydrogenase A(LDHA) increased significantly after C1q treatment (**Figures 5A, B**). CPT1α and LDHA respectively center in the process of fatty acid oxidation and glycolysis. Besides, the expression of transcription factor HIF1α, an important oxygen sensor and metabolic modulator, also increased after C1q treatment (**Figure 5C**). The data suggested that C1q treatment can increase T cell metabolism, which was confirmed by the seahorse experiment (**Figures 5D, E**). Consistent with metabolic changes aforesaid, the lactate content was found increased in Teff and Tmem internally and culture medium of Teff after C1q treatment (**Figures 5F, G**). Increased glycolysis and higher lactate production further confirmed the effects of C1q treatment on T cell metabolism. Taken together, the results suggested that Teff was considerably responsive to C1q on metabolic regulation.

Some metabolites have been proved to involve in epigenetic regulation as substrates, donors, cofactors, or antagonists of chromatin- and DNA-modifying enzymes, indicating cross-talk between cell metabolism and differentiation (27). Previous studies have found that LDHA could regulate cell differentiation through the epigenetic axis (28). We next focused on whether the alternation of epigenetic profiling occurred in CD8^+^ T cells after C1q treatment. Since lactate is generated in the cytoplasm which gives rise to acetyl-CoA and increases the level of histone acetylation, H3K27 and H3K9 acetylation levels were also elevated in both Teff and Tmem after C1q treatment (**Figures 5H, I**), which is consistent with our observation of increased lactate concentration in treated cells (**Figures 5F, G**).

### 3.6 C1q regulates T cell differentiation and function

**Figure 6:**
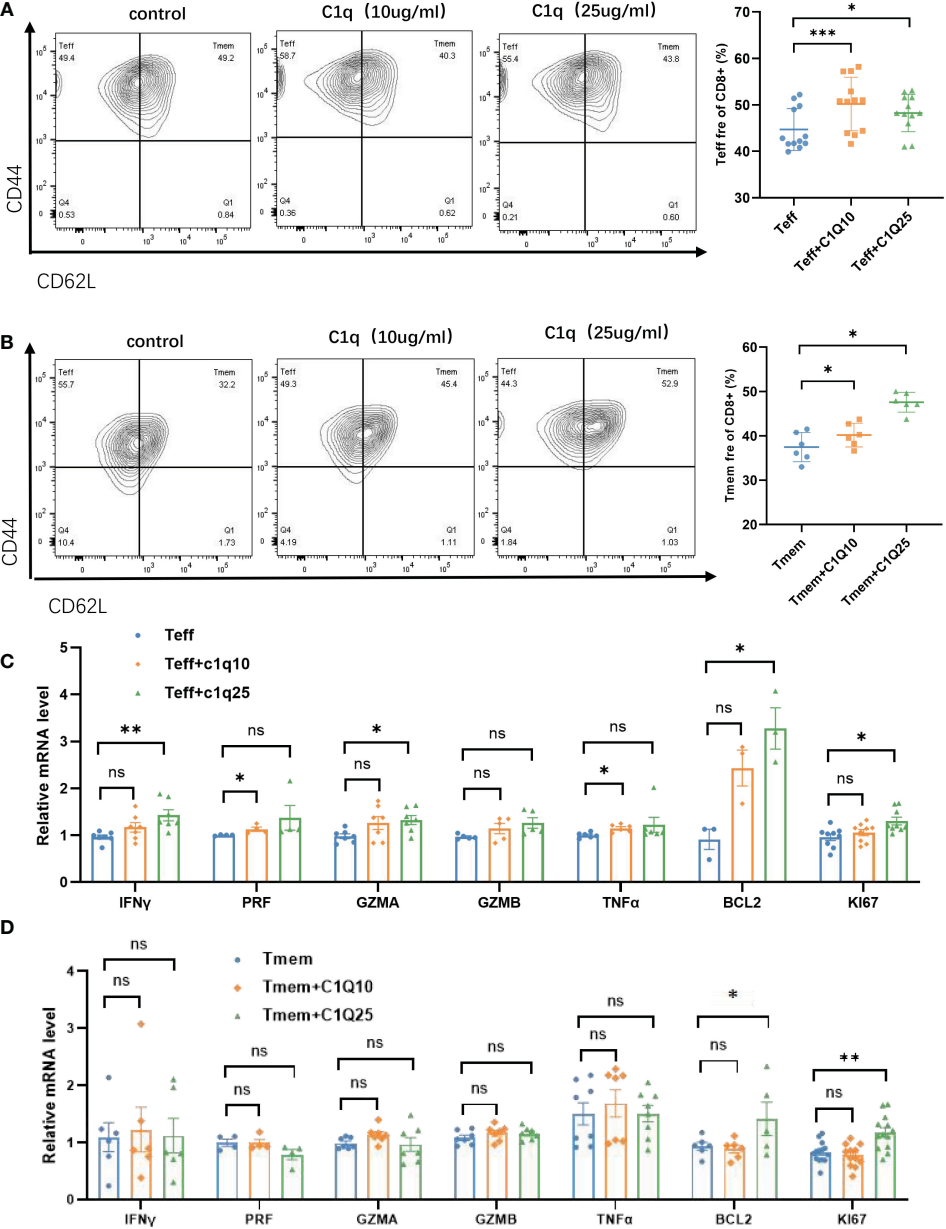
Effects of C1q on T cell differentiation and function **(A)** Flow cytometric gating of CD44 and CD62L in CD8^+^Teff (left) cells and quantification (right) on Teff cells with and without C1q. **(B)** Flow cytometric gating of CD44 and CD62L in CD8^+^Tmem (left) cells and quantification (right) on Tmem cells with and without C1q. **(C)** The expression of IFNγ, GZMA, PRF, GZMB, TNFα, BCL2, and KI67 in Teff cells with and without C1q. **(D)** The expression of IFNγ, GZMA, PRF, GZMB, TNFα, BCL2 and KI67 in Tmem cells with and without C1q. ns, no significance; *P<0.05; **P<0.01; ***P<0.001. IFNγ, Interferon Gamma, GZMA, Granzyme A, GZMB, Granzyme B, PRF, Perforin, TNFα, Tumor Necrosis Factor Alpha, BCL2, BCL2 Apoptosis Regulator, KI67, Marker Of Proliferation Ki-67.

Then we turned to the impact of C1q from C16 on T cell activation and proliferation. Naïve CD8^+^T cells were isolated from wild type mice, then stimulated by anti-CD3 and anti-CD28 antibodies, along with C1q to simulate the effect of CD3^+^C1q^+^TAM on T cells function. We found that C1q treatment promoted Teff and Tmem differentiation (**Figures 6A, B**) through T cell responses analysis by flow cytometry. Then we assessed the effects of C1q on the production of IFN-γ, tumor necrosis factor–α (TNF-α), Perforin (PRF), Granzyme A (GZMA) and Granzyme B (GZMB) in Teff and Tmem cells. For both Teff and Tmem, after C1q treatment, cells had increased expression of Ki67 and Bcl2, possibly highlighting a link between C1q and T cell proliferation and survival capacity although the effector function was only significantly enhanced in Teff (**Figures 6C, D**). The treatment did not affect PD1 or PDL1 expression of T cells, thereby excluding that C1q might induce T cell exhaustion (**Supplementary Figure 4**).

## 4 Discussion

Understanding the tumor immune microenvironment of HCC is critical for developing individualized immunotherapy. The study performed cell clustering and annotated them based on scRNA-seq from paired HCC tumor or peri-tumor tissues. Combing the TCGA data, we established the correlation between each cluster and the prognosis of liver cancer patients (**Figure 2**). Some clusters (C1, C13, C16, and C18) predicted better survival; in contrast, C3, C12, and C14 were linked to poor prognosis.

In view of the prognostic value of C16, it is conceivable that these cells play a crucial role in anti-tumor immunity. C16 is a new population simultaneously expressing CD3, C1Q, CD68, APOEFTL, SELENOP, FCER1G, and HLA-DRA (**Table 1**). CD68 is a classic marker for macrophages. Complement component C1q is a marker of a particular subpopulation of tissue-resident macrophages and tumor-associated macrophages (TAM). It is hard to define this population as M1(pro-inflammation) or M2(anti-inflammation) based on their DEGs generated from scRNA-seq data. Then we verified its entity by staining well-known markers for M1 (CD80, CD86) and M2(CD206, CD163) signaling, and the results suggested it is a unique population of cells that possess both M1 and M2 features.

With high plasticity, the differentiation process of macrophages often produces heterogeneous results. Recently, a novel subset of macrophages called CD3^+^ macrophages was found accumulating in both infectious and non-infectious condition (23).They have been reported to originate from monocytic myeloid-derived suppressor cells (M-MDSCs) (29). Meanwhile, CD3^+^monocyte-derived macrophages can be further differentiated into TCRαβ^+^ and TCRαβ^−^ sub-population. Adriana et al. found that Tumor necrosis factor (TNF) knockout caused lower frequency of CD3^+^ TCRαβ^+^ macrophage and higher mortality in Bacillus Calmette-Guérin (BCG) -infected mice (23). Consistently, the ϵ and ζ chains of CD3 were recently reported to express on RAW, a classical murine macrophage cell line, and present increased transcriptional levels after BCG infection (30). In this study, we identified CD3^+^C1q^+^macrophages, a new subset in both HCC tumors (**Figure 1**) and peripheral blood of sepsis patients (**Figure 4**). In sepsis samples, the population of CD3^+^C1q^+^macrophages had a sharp rise at Day1 after infection and subsequent decline when the infection subsided (**Figure 4A**). So, we believe that the tumor-infiltrated CD3^+^C1q^+^TAM mainly plays a pro-inflammatory and anti-tumor role in the initial stage of infection and tumors although the population is experiencing differential and heterogeneous characteristics. It also could explain why the increased CD3^+^C1q^+^TAM links to a better survival prognosis for liver cancer patients (**Figure 2E**).

We quantified the interaction information between clusters for visual analysis. The results showed that the cluster-cluster interactions differed in HCC tumors and para-cancerous tissue. To further understand the transcriptional heterogeneity of clusters, we discovered transcription factors related to the oncogenesis and development of HCC. For C6 and C16, the gene regulation network of specific high expression transcription factors was constructed. Some key transcription factors were found, such as KDM5B, UQCRB, and CREM in C6, at the same time DAB2 in C16(data not shown).

It has been reported that C1q^+^TAMs could modulate tumor-infiltrating CD8^+^T cells *via* multiple immunomodulatory ligands (31). However, in our study the new identified CD3^+^C1q^+^TAM hasn’t been reported before. Therefore, the explanation for the relationship between newly identified C16(CD3^+^C1q^+^TAM) and C6 (CD8^+^CCL4^+^T) cells also remains unknown. Based on multiple roles of C1q independent of complement activation (32), we explored how CD3^+^C1q^+^TAM affected the CD8^+^CCL4^+^T cells.

*In vitro* experiment suggested that this population can promote specific T cell functions by some mechanisms independent of complement activation. In the study (**Figure 5**), C1q treatment affected multiple metabolic genes expression and which was confirmed by a seahorse experiment. Besides, an abundant metabolite produced during glycolysis, lactate, was increased along with enhanced histone acetylation in both Teff and Tmem after C1q treatment (**Figures 5H, I**). In other studies, complement C1q binding protein (C1qbp) has been reported to promote differentiation of Teff *via* metabolic-epigenetic reprogramming (33). Besides, research showed that C1q could restrain responses to self-antigens in CD8^+^T cells by modulating mitochondrial metabolism (34). Altogether these data link C1q to CD8^+^T cell metabolism and activation and point the protective role of this newly identified population. Dynamic changes of histone acetylation lead to differential expression of effectors, facilitating rapid and robust responses in CD8^+^Tmem (35). The nature of incoming environmental factors and the diversion of inherent metabolic programs during T cell activation heavily impact the differentiation and function of effector T cell subpopulations. Thus, our findings depict how C1q influences the metabolism of CD8^+^T cells and highlights the importance of interaction between complement and immunometabolism in anti-tumor immunity.

With the development of gene detection technology, genomic network-based stratification (NBS)-guided treatments have made great progress clinically. Tumor mutations were proved to help identify subtypes in ovarian, uterine, endometrial and lung cancer and head and neck squamous cell carcinoma (HNSCC) (36–39). A metabolic network-driven approach facilitated to divide HCC into 3 subsets with distinct metabolic features and different prognosis (39). Besides, transcription regulators were also reported to label immunoreactive or silent subtypes in cancer (40). Genomic directed stratification made it possible to implement personalized medicine. Our data showed that C16 was significantly associated with patient outcomes. Together, our results identify C1q^+^CD3^+^TAM cells relate with cancer patients prognosis and highlight these cells, as well as C1q itself, as potential targets to address TME-associated immune dysfunction. One of the limitations of our study is the lackness of *in vivo* animal model to evaluate whether T cell function can regain after restoration of the attenuate crosstalk(C16-C6) within HCC TME. Besides, more researches await to be done to explore how to strengthen this connection pointedly in clinical practice.

## Data availability statement

Data transparency. The datasets used or analyzed during the current study are available from the corresponding author on reasonable request. The data presented in the study are deposited in the China National Center for Bioinformation / Beijing Institute of Genomics, accession number HRA003563 (https://ngdc.cncb.ac.cn/gsa-human).

## Ethics statement

The studies involving human participants were reviewed and approved by Zhongshan Hospital ethics committee. The patients/participants provided their written informed consent to participate in this study. The animal study was reviewed and approved by Zhongshan Hospital ethics committee.

## Author contributions

YY designed and conducted the research, collected and interpreted the data, and wrote the paper. LS, ZC, FL, MM, YC, YL performed and assisted with the experiments, helped to analyze the data and edit the manuscript. QX and GC performed scRNA-seq data analysis. WL, HF and YS provided the patient samples and assisted in the analysis of experimental results. LS, FL, WL, QX, MM, YC, YL, GC and YS provided advice and edited the paper. DW oversaw the research and revised the paper. The authors declared that they had no conflicts of interest. All authors contributed to the article and approved the submitted version.

## Supplementary material

Supplementary Figure 1 Differentiation and stemness potential analysis of CD4 and CD8 cell subsets.

Supplementary Figure 2 Flow cytometry gating of M1 (CD80^+^ CD86^+^), M2 (CD163^+^ CD206^+^) staining on C1q-TAM (CD11B^+^ CD68^+^) cells in HCC.

Supplementary Figure 3 Effects of C1q on T cell metabolic genes expression.

Supplementary Figure 4 Effects of C1q on T cell exhaustion induction.

Supplementary Table 1 Primer sequences of genes related to glycolysis and effector functions are provided.
