# Supplementary figures and images for: The immune-metabolic crosstalk between CD3+C1q+TAM and CD8+T cells associated with relapse-free survival in HCC
Source: Front Immunol. 2023 Feb 9;14:1033497. doi: 10.3389/fimmu.2023.1033497 (PMC9948089; doi:10.3389/fimmu.2023.1033497)

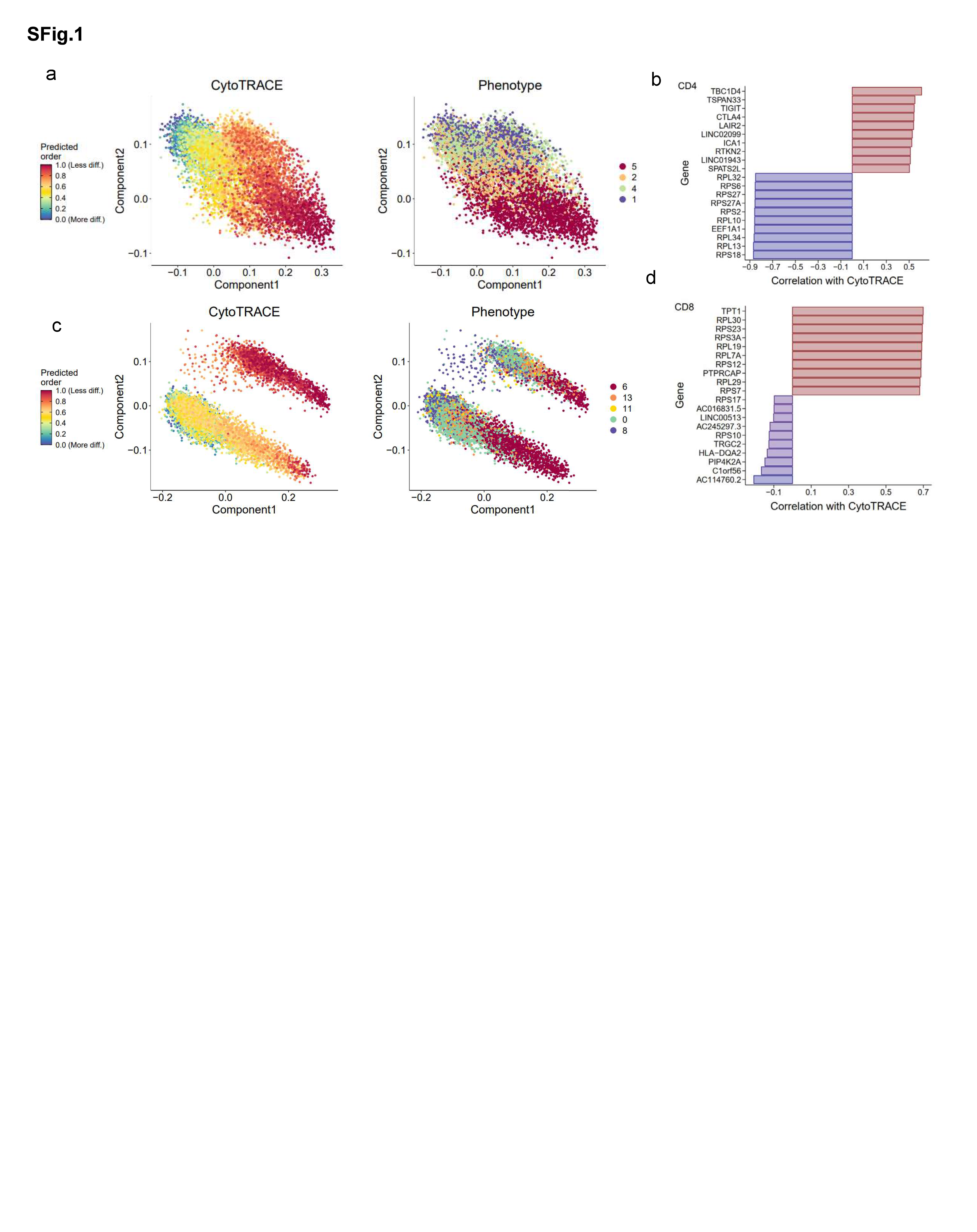

Supplement: Supplementary Figure 1 — Differentiation and stemness potential analysis of CD4 and CD8 cell subsets. [file Image_1.tif]

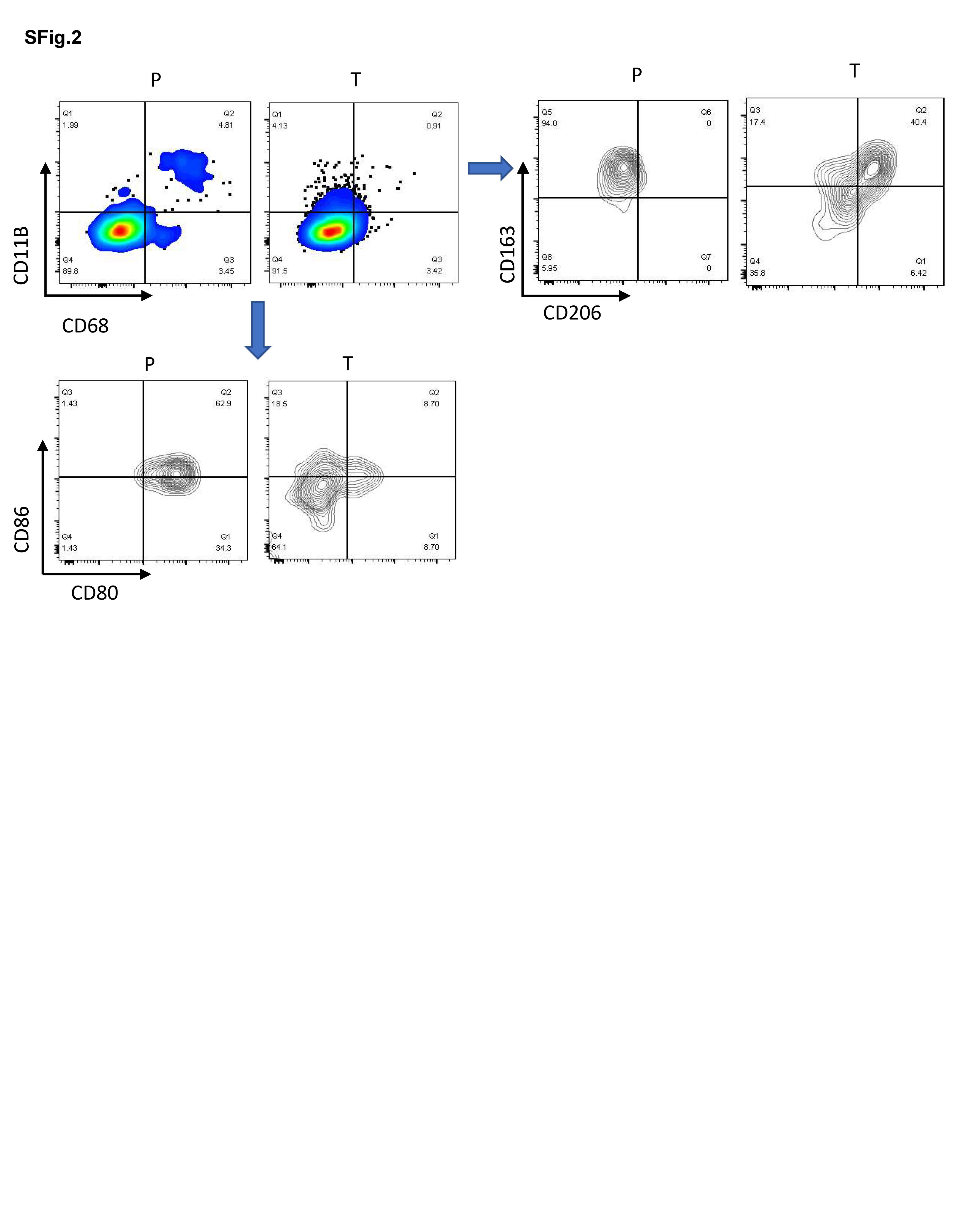

Supplement: Supplementary Figure 2 — Flow cytometry gating of M1 (CD80+ CD86+), M2 (CD163+ CD206+) staining on C1q-TAM (CD11B+ CD68+) cells in HCC. [file Image_2.tif]

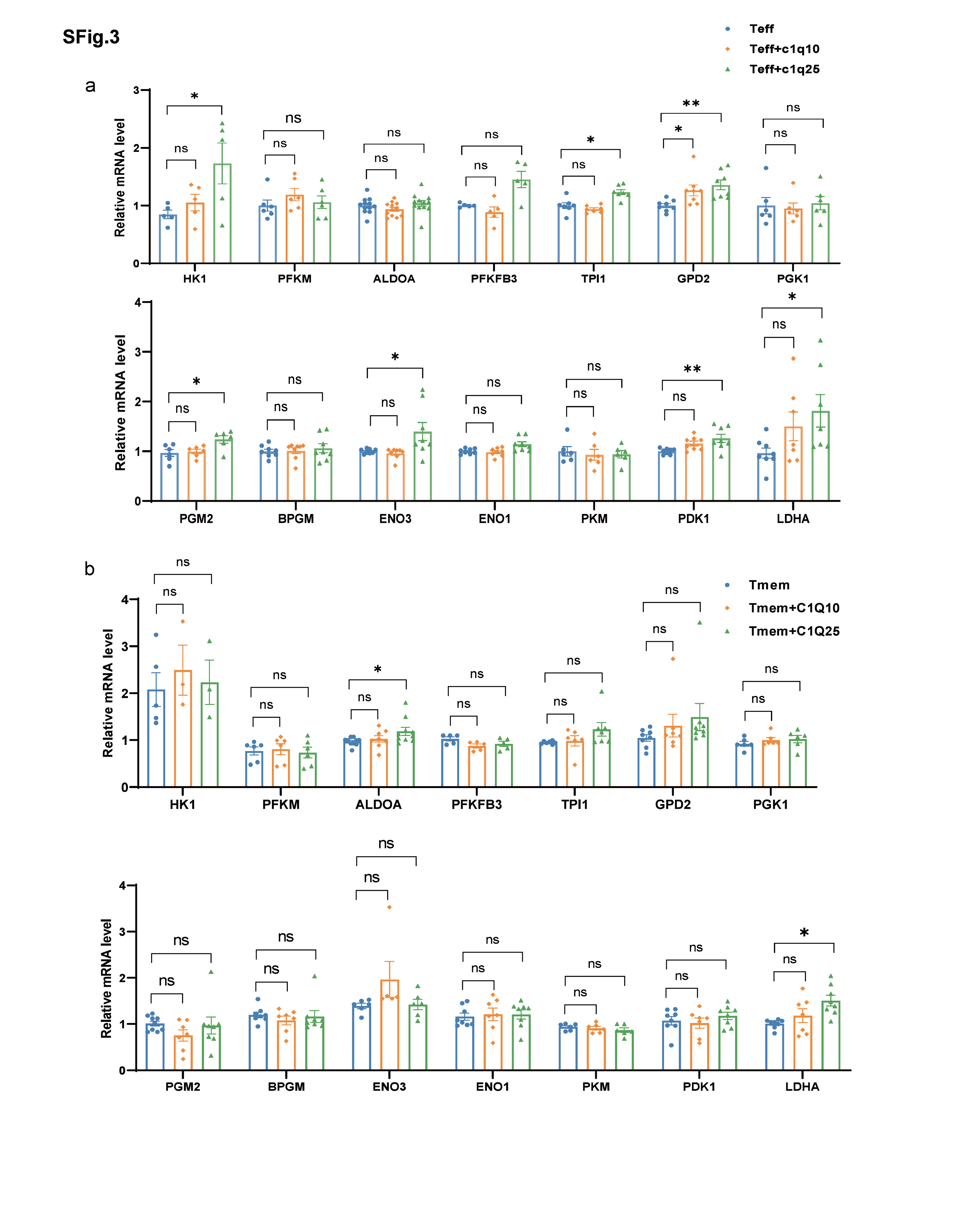

Supplement: Supplementary Figure 3 — Effects of C1q on T cell metabolic genes expression. [file Image_3.tif]

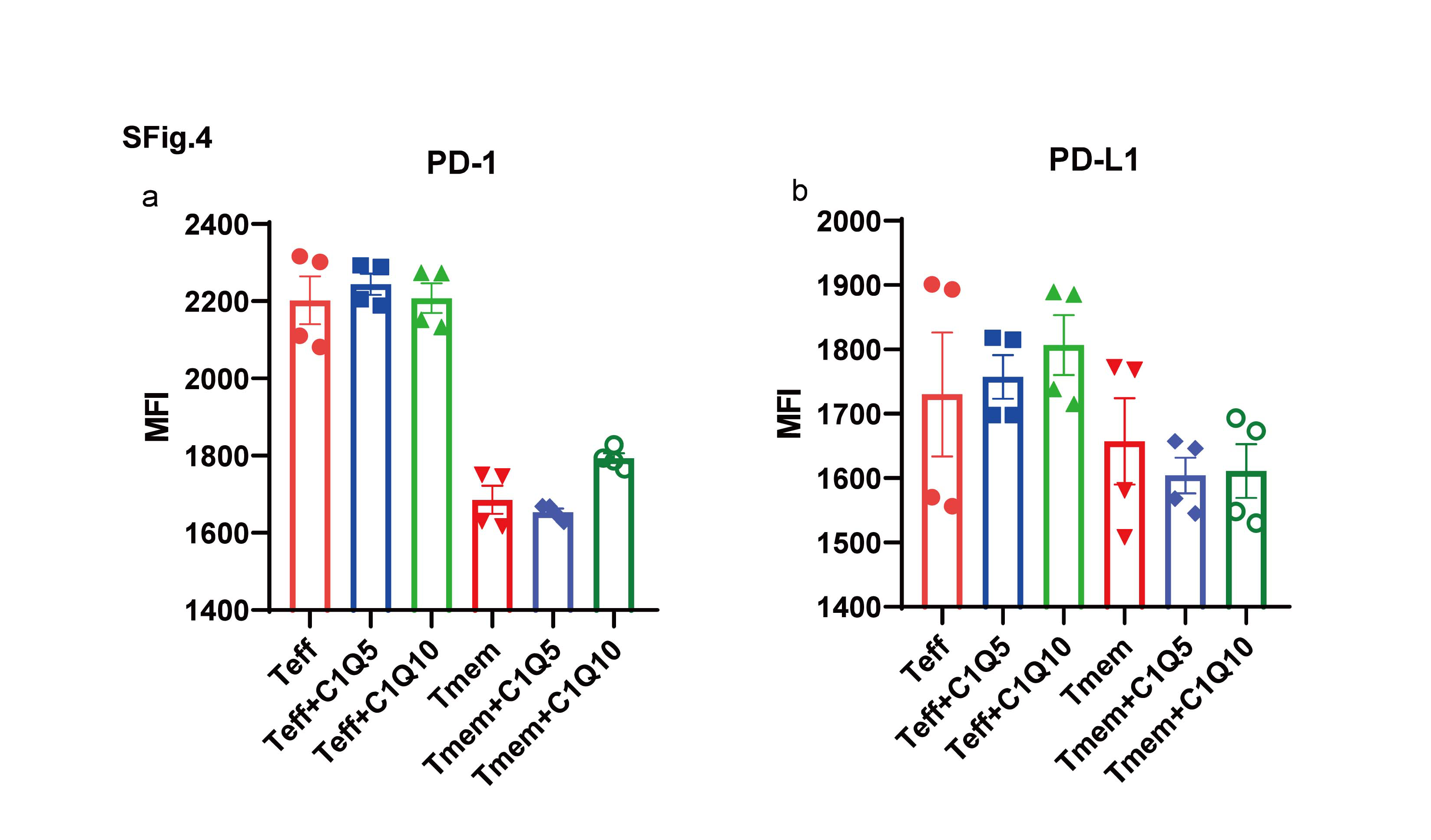

Supplement: Supplementary Figure 4 — Effects of C1q on T cell exhaustion induction. [file Image_4.tif]
