# Supplementary material for: The immune-metabolic crosstalk between CD3+C1q+TAM and CD8+T cells associated with relapse-free survival in HCC
Source: Front Immunol. 2023 Feb 9;14:1033497. doi: 10.3389/fimmu.2023.1033497 (PMC9948089; doi:10.3389/fimmu.2023.1033497)
Supplement: Supplementary Table 1 — Primer sequences of genes related to glycolysis and effector functions are provided. [file Table_1.docx]

Supplementary table 1: Primer sequences of genes related to glycolysis and effector functions are mentioned in the text

| Name |  | Sequence (5' to 3') |
| --- | --- | --- |
| mus-36B4 | Forward | AGATTCGGGATATGCTGTTGGC |
|  | Reverse | TCGGGTCCTAGACCAGTGTTC |
| mus-HK1 | Forward | AGGGCGCATTACTCCAGAG |
|  | Reverse | CCCTGTGGGTGTCTTGTGTG |
| mus-PFKM | Forward | CGTGGGAGAGCGTGTCTATGA |
|  | Reverse | CTCGCTCTCGGAAGTCCTTG |
| mus-Aldoa | Forward | AATGTTCTGGCCCGTTATGC |
|  | Reverse | GGAGAATTTCAGGCTCCACAAT |
| mus-PFKFB3 | Forward | CCCAGAGCCGGGTACAGAA |
|  | Reverse | GGGGAGTTGGTCAGCTTCG |
| mus-TPI1 | Forward | CAAACCAAGGTCATCGCAGA |
|  | Reverse | GCCCACACAGGTTCATAGGC |
| mus-GPD2 | Forward | GAAGGGGACTATTCTTGTGGGT |
|  | Reverse | GGATGTCAAATTCGGGTGTGT |
| mus-PGK1 | Forward | GCTGTTCTCCTCTTCCTCATC |
|  | Reverse | CCTTTGGTTGTTTGTTATCTGG |
| mus-PGM2 | Forward | AGTGAAGACGCAGGCATATCC |
|  | Reverse | GGCTCCACGGTAGAGACGA |
| mus-Bpgm | Forward | CTCCTGAAACATCTGGAAGGTATCTC |
|  | Reverse | CAGTGGGCAGAGTGATGTTGAT |
| mus-Eno1 | Forward | TGCGTCCACTGGCATCTAC |
|  | Reverse | CAGAGCAGGCGCAATAGTTTTA |
| mus-Eno3 | Forward | CGACACATCGCAGATCTTGC |
|  | Reverse | CCGTTGATCACATTAAAGGCAG |
| mus-PKM | Forward | GCCGCCTGGACATTGACTC |
|  | Reverse | CCATGAGAGAAATTCAGCCGAG |
| mus-PDK1 | Forward | GGACTTCGGGTCAGTGAATGC |
|  | Reverse | TCCTGAGAAGATTGTCGGGGA |
| mus-LDHA | Forward | TGTCTCCAGCAAAGACTACTGT |
|  | Reverse | GACTGTACTTGACAATGTTGGGA |
| mus-HIF1a | Forward | ACCTTCATCGGAAACTCCAAAG |
|  | Reverse | CTGTTAGGCTGGGAAAAGTTAGG |
| mus-BCL2 | Forward | GTCGCTACCGTCGTGACTTC |
|  | Reverse | CAGACATGCACCTACCCAGC |
| mus-IFNγ | Forward | ATGAACGCTACACACTGCATC |
|  | Reverse | CCATCCTTTTGCCAGTTCCTC |
| mus-TNF | Forward | CCCTCACACTCAGATCATCTTCT |
|  | Reverse | GCTACGACGTGGGCTACAG |
| mus-PRF | Forward | AGCACAAGTTCGTGCCAGG |
|  | Reverse | GCGTCTCTCATTAGGGAGTTTTT |
| mus-GZMA | Forward | TGCTGCCCACTGTAACGTG |
|  | Reverse | GGTAGGTGAAGGATAGCCACAT |
| mus-GZMB | Forward | CCACTCTCGACCCTACATGG |
|  | Reverse | GGCCCCCAAAGTGACATTTATT |
| mus-KI67 | Forward | ATCATTGACCGCTCCTTTAGGT |
|  | Reverse | GCCTCGCCTTGATGGTTCCT |
